# Studying molecular interactions in the intact organism: fluorescence correlation spectroscopy in the living zebrafish embryo

**DOI:** 10.1007/s00418-020-01930-5

**Published:** 2020-10-16

**Authors:** Michael L. Dawes, Christian Soeller, Steffen Scholpp

**Affiliations:** 1 Living Systems Institute, School of Biosciences, College of Life and Environmental Sciences, University of Exeter, Exeter, EX4 4QD UK; 2 Living Systems Institute, College of Engineering, Mathematics, and Physical Sciences, University of Exeter, Exeter, EX4 4QD UK

**Keywords:** Microscopy, Protein–protein interactions, Fluorescent correlation spectroscopy (FCS), Zebrafish

## Abstract

Cell behaviour and function is determined through the interactions of a multitude of molecules working in concert. To observe these molecular dynamics, biophysical studies have been developed that track single interactions. Fluorescence correlation spectroscopy (FCS) is an optical biophysical technique that non-invasively resolves single molecules through recording the signal intensity at the femtolitre scale. However, recording the behaviour of these biomolecules using in vitro-based assays often fails to recapitulate the full range of variables in vivo that directly confer dynamics. Therefore, there has been an increasing interest in observing the state of these biomolecules within living organisms such as the zebrafish *Danio rerio*. In this review, we explore the advancements of FCS within the zebrafish and compare and contrast these findings to those found in vitro.

## Introduction

Determining the behaviour of molecules requires precise measurements within their complex environments that are in constant interplay with multiple factors. Much of our understanding of protein dynamics is due to measurements in homogenous solutions. These experiments precisely determine the dynamics within the environment of that particular solution but fail to recapitulate the complex environment of a cell, a tissue or ultimately an organism in vivo. These environments influence molecular behaviour in various ways such as constraints imposed by cellular compartments and their respective constituents (Wenger et al. 2007). At the subcellular level, these environmental influences change significantly over short distances between the cytosol, plasma membrane and extracellular space to name a few. Specific protein–protein interactions, co-factors and post-translational modifications among others further alter molecule dynamics. Examples of these modifications are the changes observed in diffusing ligands when bound within protein complexes or the alteration to binding affinities of phosphorylated proteins (Müller et al. 2013; Raman et al. 2007). In vitro assays lay the foundations for protein kinetics, but there remains the crucial need for re-examination within the natural biological environment in vivo.

**Fig. 1:**
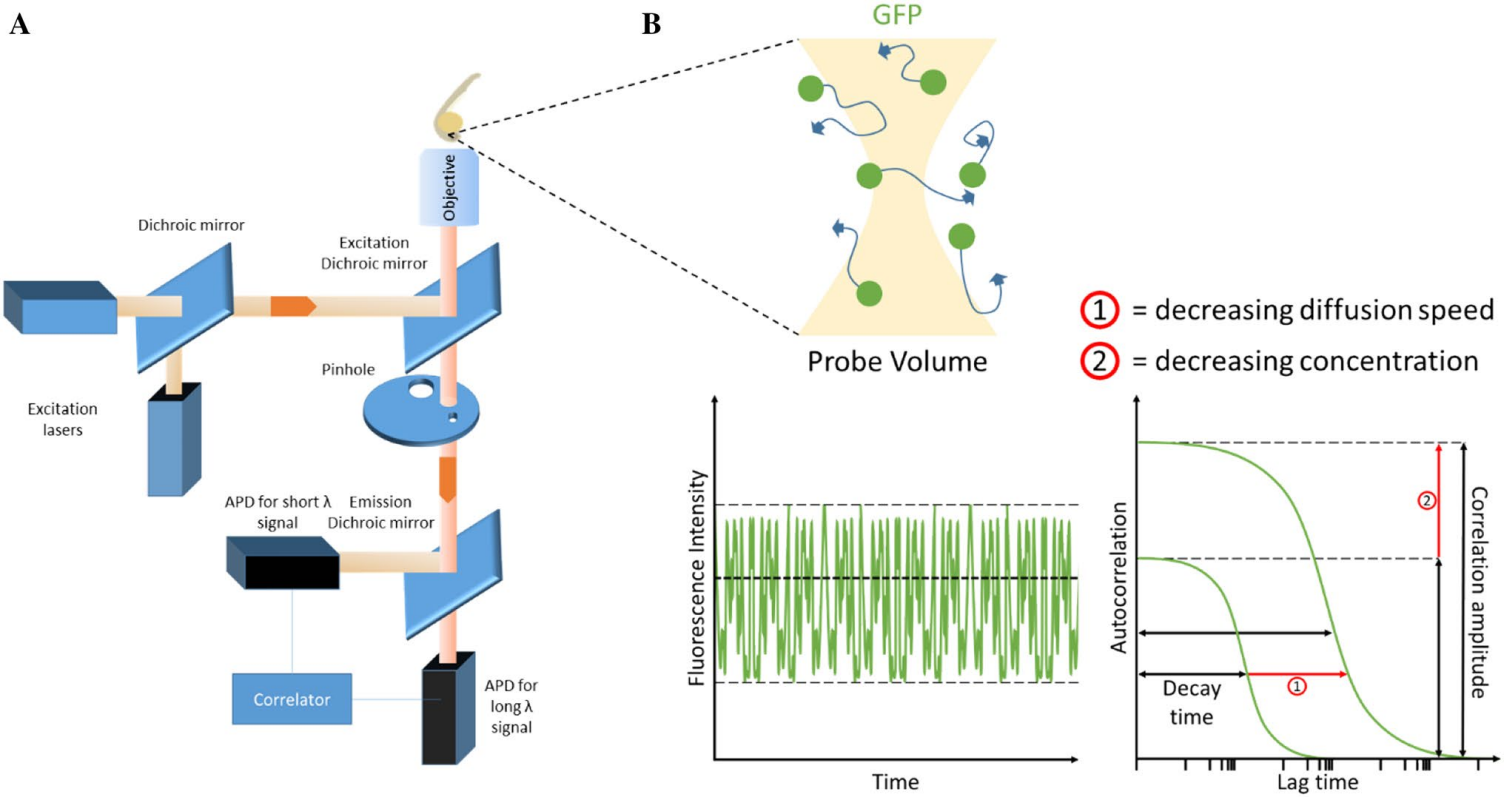
Overview of FCS set-up and zebrafish measurements. **a** A schematic of a typical FCS set-up. Single or dual excitation lasers can be configured to excite one or two types of fluorophores for FCS and fluorescence cross-correlation spectroscopy (FCCS), respectively. Dichroic mirrors are in place to split and/or reflect beam paths of certain wavelengths. The excitation beam is sent through the objective to excite fluorophores in a zebrafish embryo. Emission is detected back along the same optical path and through the excitation dichroic mirror. Long- and short-wavelength emissions are split by an emission dichroic mirror to allow avalanche photodiodes (APDs) to detect specific fluorophores. **b** Cartoon of probe volume that FCS laser beam passes through. The probe volume excites diffusing green fluorescent protein (GFP) and receives an emission spectrum. Emission beam signal intensity is recorded over time and transformed via a suitable fitting model into interpretable data that details the sample’s diffusion coefficient and concentration. (1) Reduced diffusion speed, (2) reduced concentration

A technique that captures and resolves single molecular dynamics is fluorescence correlation spectroscopy (FCS) (Fig. 1). FCS is a correlation analysis of fluctuations of the fluorescence intensity emitted from fluorophore ligated molecules (Magde et al. 1972, 1974; Elson and Magde 1974). Similar to confocal microscopy, FCS emits an excitation beam to stimulate the fluorophores within the sample and receive their respective emission spectra past a pinhole for analysis. The significant difference between the two techniques is that FCS precisely excites a focal volume of a few femtolitres to resolve individual fluorophores. Fundamentally, all molecules are subject to random fluctuations as a result of Brownian motion, but the fluctuations in the recorded signal as molecules move in and out of the probe volume carry fingerprints of the properties of the individual molecules. FCS therefore determines the parameters of the fluorophore such as protein diffusion kinetics and concentration as a function of their emission spectra fluctuations. This technique is unique from other biochemical tracing techniques as it relies on optics alone, therefore allowing non-invasive spatiotemporal analysis of intact cells or even tissues.

The principle of FCS is based on the idea that the statistical analysis of the fluorescence emission fluctuations resulting from fluorescently labelled proteins of interest (POIs) entering and leaving a typically stationary probe volume (Fig. 1a) can be used to reveal characteristic properties of these POIs. Properties of interest include their mobility (quantified, for example, by the diffusion coefficient), their concentration and also stabilities or binding affinities when pairs of interacting proteins are studied in cross-correlation analysis. The extraction of these parameters of interest typically requires the fitting of biophysical model equations to the experimentally obtained correlation spectra which is technically complex in its own right (Fig. 1b). A number of reviews have focused on these technical aspects of FCS (Elson 2011); here, we will instead focus on higher-level questions relating to the appropriateness for biological interpretation of the environments in which FCS experiments are carried out and only mention the complexity of interpreting FCS data where needed.

There remain prerequisites to the sample in preparation for FCS such as the addition of a fluorescent tag on the molecule or POI. Despite the direct modification of the target molecule and the potential perturbation associated with it, FCS provides the advantage of observing a specific molecule in a living organism and observes changes of its behaviour in its appropriate environment. While capable of observing physiologically relevant environments, FCS also allows multiple repeated measurements of the same sample over time. This is particularly powerful in observing developing models as various aspects of the sample environment and molecular characteristics are subject to change.

But, all that glitters is not gold as FCS, like any measurement technique, also comes with specific technical limitations that are important to consider in practice. The main disadvantage of FCS is that the POI needs to fluoresce. This can be achieved by adding fluorescent tags to the biological protein; however, one needs to ensure that the tag itself does not modify the property of interest of the molecule too greatly. Furthermore, measurement of fast moving particles is preferable because otherwise photobleaching can eventually occur, a phenomenon that can be partially combated with pre-bleach treatment of the sample or post-analysis correction (Ries et al. 2010). Finally, the analysis of single molecule behaviour in a complex cellular environment can be challenging. The analysis might require complex curve fitting algorithm and low-quality detection of the fluorophores that can cause ambiguity of the data set.

In this review, we will specifically discuss FCS as a method to measure, quantify and interpret protein kinetics in a living zebrafish embryo. First, we will describe the usage of this technique in comparison with more traditional in vitro approaches. Then, we will explain the advantage of zebrafish embryos as a model organism for these fluorescence-based protein–protein dynamics studies. We will further elucidate more practical aspects important to consider when using zebrafish with FCS. Finally, we will give an outlook and discuss technical advances required to make use of the full potential of FCS in the life sciences.

### FCS and the eternal battle between in vitro and in vivo

FCS was first conceived in 1970 to determine the molecular processes that influence fluorescent fluctuations in a small observation volume in solution (Magde et al. 1972). FCS was implemented to observe chemical reaction rates, molecular mobility (diffusion coefficients and flow velocities), particle sizes and concentrations, as well as molecular aggregation and interactions (Krichevsky and Bonnet 2002; Haustein and Schwille 2007; Ries and Schwille 2012). This made FCS particularly attractive in biological fields as it can resolve the chemical behaviour of molecules and POIs. Naturally, in many early studies POIs were isolated and suspended in a homogenous solution for in vitro FCS analysis. These studies generated a plethora of valuable information on factors such as enzyme reaction kinetics (Heinze et al. 2002), protease activity (Kohl et al. 2005) and protein diffusion for the understanding of transport or morphogen gradients (Dittrich and Schwille 2002). Enzyme kinetics have been elucidated by cross-correlating the emission of two spectroscopically distinct fluorophores in an adaptation of FCS called fluorescent cross-correlation spectroscopy (FCCS). Correlation of the emission spectra from the enzyme and substrate measure not only their dissociation constant but the rate of substrate breakdown (Lee et al. 2011).

**Fig. 2:**
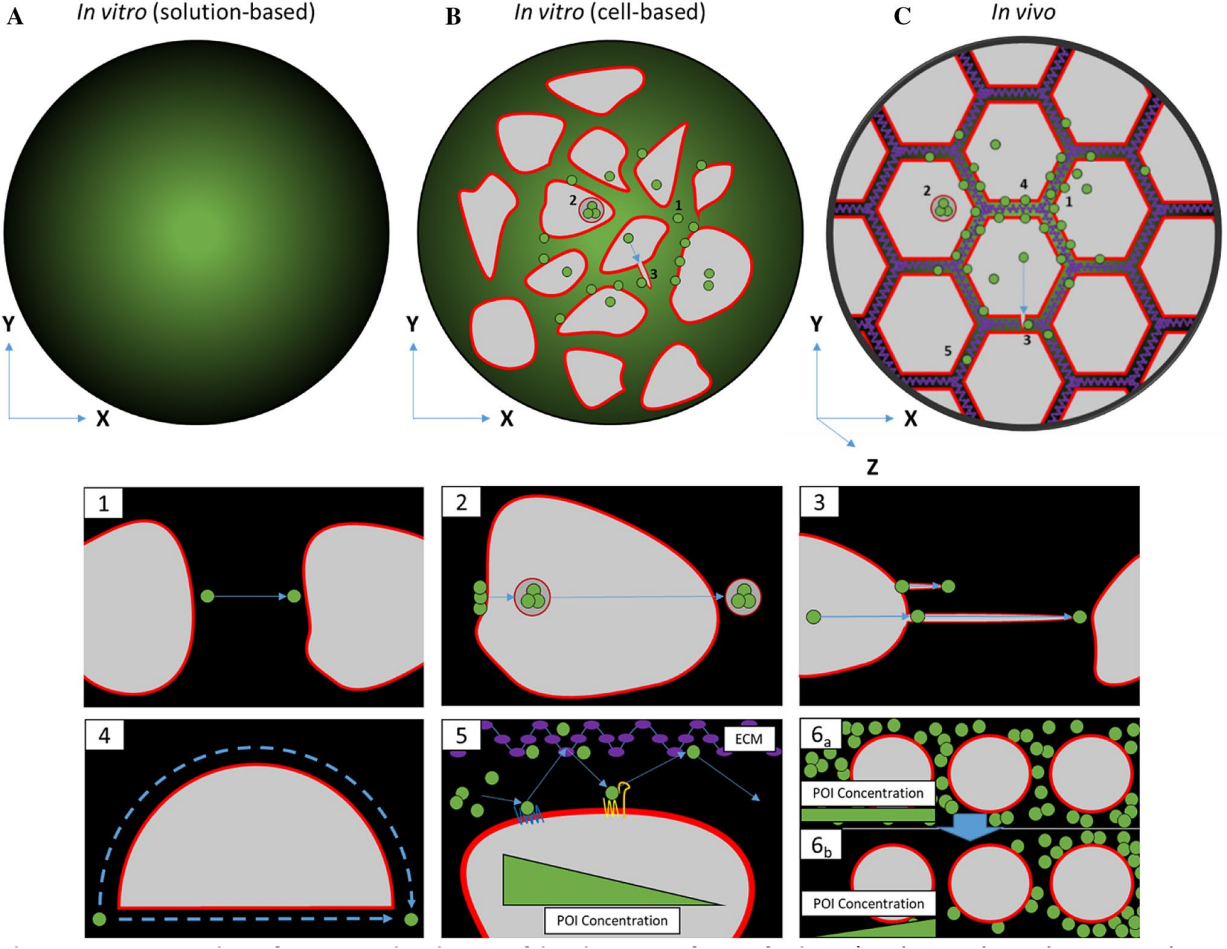
Comparison between in vitro and in vivo sample analysis. **a** Solution-based assays rely on simple diffusion in an essentially homogenous solution. **b** Cell-based in vitro assays allow analysis of dynamics of intracellular processes such as (1) extracellular diffusion, (2) transcytosis and (3) the formation of signalling filopodia such as cytonemes. **c** In vivo (including three-dimensional collagen/matrigel cell-based) assays involve all aspects observed in cell-based in vitro assays and further parameters of molecular diffusion such as (4) tortuosity (hindrance of diffusion path by impermeable objects), (5) transient binding of molecules to the extracellular matrix (ECM) and the cell membrane, and (6) restrictive clearance for gradient formation and the additional *Z*-axis that introduces further dimensions for diffusion

FCS measurements using in vitro solutions provide rapid and robust results that are easily scalable for observing a large range of proteins (Fig. 2a) (Wood et al. 2011; Lange et al. 2013; Wachsmuth et al. 2015). Despite these technical advantages, solution-based FCS has been shown to poorly reflect the in vivo dynamics of proteins and molecules for a number of reasons. There is a wide range of biotic and abiotic variables present in a cellular environment that are missing in solution-based preparations—from multiple tissue types down to the cellular level. Multiple factors including cell compartments that constrain motion and complex microenvironments affect protein dynamics in ways that can hardly be recapitulated in solution-based FCS (Fig. 2b). The binding affinity between DKK1 and Kremen2, a ligand and cell surface receptor, respectively, that functions to regulate Wnt signalling pathway, serves an example of this dilemma (Dörlich et al. 2015). In vitro studies identified that Kremen2 cooperates with DKK1 to stabilise binding to LRP6 and therefore promote LRP6 turnover with a ratio of unbound to bound protein concentration being of < 3 nM, otherwise denoted by the dissociation constant (*K*_D_ = concentration of unbound protein/concentration of bound protein). Repeating the experiment in a cell-based setting revealed the similar dynamics but at a significantly, at least threefold, reduced affinity with a higher K_D_ of 10.3 ± 2.1 nM. The discrepancy observed between the two *K*_D_ values highlights various unforeseen factors—in this case most likely the requirement of modifying co-factors—that play a role in the interaction between the two proteins*.*

### The model organism zebrafish in imaging-based analysis

It is in the interest of every biological investigation to capture molecular dynamics within the context of a physiologically relevant environment. Therefore, in vivo studies are regarded as the ‘gold standard’ in observing protein dynamics within the context of a physiologically relevant background (Fig. 2c). To achieve the closest relevance, researchers develop new methods for investigating their POI within living organisms that are compatible with FCS measurements. However, measuring protein kinetics in an intact organism comes with a myriad of challenges that have been explored and now partially or fully overcome for a variety of model organisms. One challenge for optical-based studies is the introduction of artefacts within the emitted signal as a result of sources of contaminating background signals from the organisms’ tissues. Auto-fluorescent constituents within the sample, i.e., proteins or small molecules that contribute a background signal, may be difficult to distinguish from the POI fluorescence and can mask the fluctuations of the POIs themselves. Furthermore, the heterogeneous optical refractive indexes of cells and tissue hinder the maximum penetration depth over which FCS can be performed. Accordingly, measurements can suffer from low signal-to-noise ratio that make parameter extraction difficult or even impossible (Shi et al. 2009a, b). Therefore, only a few model organisms permit the unimpeded study with FCS as most suffer to a variable extent from the technical problems described above. Model organisms ranging from invertebrates, such as *Caenorhabditis elegans* (Beam et al. 2012) and *Drosophila melanogaster* (Wang et al. 2004), to vertebrates like *Xenopus laevis* and the zebrafish *Danio rerio* have all been studied with FCS (Shi et al. 2009a, b). The zebrafish, in particular, has greatly furthered the field of developmental biology with the use of FCS and other confocal-microscopy-related quantification technologies such as FCCS, Förster resonance energy transfer (FRET) and fluorescence recovery after photobleaching (FRAP) (Deng et al. 2020; Wang et al. 2019).

The zebrafish is a powerful model organism for a number of reasons. The zebrafish holds a close genetic composition to that of humans with approximately 70% of human genes sharing at least one orthologue in zebrafish (Howe et al. 2013). Furthermore, the zebrafish exhibits a desirable life cycle as the fish procreates every morning in response to light–dark stimuli, generating an average of over hundreds of eggs per female (Spence et al. 2006). The eggs themselves develop rapidly, generating larva with fully functional organs 48 h post-fertilisation (hpf) onwards and an independently eating juvenile 5 days post-fertilisation (dpf) with a highly recognisable and well-characterised development during the various stages of gastrulation and embryo development ( Kimmel et al. 1995). Zebrafish embryos also allow for a wide range of genetic manipulation at all stages which provides a powerful platform for developmental biology. The zebrafish genome has been heavily investigated along with a variety of genomic resources including databases of expressed sequence tags, high-density genetic linkage and radiation hybrid maps (Amemiya et al. 1999; Clark et al. 2001; Geisler et al. 1999; Shimoda et al. 1999; Vogel 2000).

The crowning advantage that distinguishes zebrafish embryos is that they develop externally and exhibit a near complete transparency of the embryonic tissue within the first 48 h. During that stage, the cells have a very low yolk content and are located around the central yolk cell, adding two parameters to the imaging qualities: short imaging depths and increased translucency of the specimen. These desirable qualities of the zebrafish have spring boarded its popularity within the field, and it has been a sought-after candidate for a variety of imaging-based studies.

### FCS analysis in zebrafish

The earliest FCS investigation using zebrafish was used to measure the flow velocities within developing organs (Pan et al. 2007). In standard FCS, the probe volume is rotationally symmetric in the focal plane which can measure the counts of fluorophores traversing the probe volume but cannot determine an overall direction of flow. A novel adaptation of FCS was devised to resolve this issue by generating a rectangular or elliptical laser beam cross section to break the symmetry of the probe volume in the plane of focus (Lenne et al. 2002). This advanced technique was applied to investigate vasculogenesis within the developing liver of the zebrafish (Korzh et al. 2008). FCS precisely identified the initiation of blood flow within the liver bud (72–75 hpf) and correlated this event with significant liver bud expansion and convergence to the sinusoids (84–120 hpf). When taken together, liver development was observed to be multi-step with the initiation of blood flow being the final and crucial step in liver development. FCS in this study allowed pinpointing the stage of blood flow initiation and correlated this event with the other developmental milestones, allowing precise determination of liver stage growth. The analysis was limited to a scanning depth of 70–80 µm as a result of optical hindrance through the zebrafish tissue, which was not sufficient to cover the entire growing liver. A maximal scanning depth of < 100 µm seems to be a current limitation of FCS in zebrafish, which occurs as a repeating consideration throughout FCS work in vivo (Korzh et al. 2008), a constraint which may require adaptive optics techniques (Ries et al. 2009) to overcome.

### FCS in morphogen transport in vivo

A major application of the FCS technology is characterisation of the behaviour of fast-moving proteins in the specimen such as chemical signalling proteins in the extracellular space. Morphogens are signalling proteins which orchestrate tissue development during embryogenesis and regeneration. Depending on their concentrations, morphogens elicit a specific response in the target cells, leading to a concentration-dependent tissue patterning process (Rogers and Schier 2011). As a superior method to measure the dynamics of morphogens and their concentrations at specific points within a cell or in a tissue, FCS has been used to understand and quantify properties of chemical gradients in tissue patterning.

Fibroblast growth factors (Fgfs) are secreted proteins that play important roles in regulating cell migration, proliferation and differentiation in vertebrate development (Böttcher and Niehrs 2005). Fgfs were among the first morphogens to be investigated within the zebrafish embryo by FCS in 2009 (Yu et al. 2009). Previously, it was unknown which factors determined the spread and range of the morphogen gradient as many suggested models such as simple diffusion and spatially uniform degradation (Crick 1970), receptor-aided bucket-brigade mechanism (Kerszberg and Wolpert 1998) and directed transport (Gregor et al. 2007) were proposed. A further model proposes that depletion of the Fgf morphogen by continuous uptake and degradation of the morphogen in the receiving cells generates a robust gradient, which has been termed restrictive clearance model (RCM) (Scholpp and Brand 2004). Consistently, by directly measuring the concentration and diffusion of Fgf8-GFP by FCS, it was determined that its respective gradient was formed passively through the generation, diffusion and receptor-mediated endocytic removal of the ligand by target cells (Yu et al. 2009). This mechanism supported the RCM with added parameters in a model defined as the ‘source–sink mechanism with freely diffusing molecules’. In concert, the family of nodal morphogens were observed to generate and maintain a morphogen gradient in a similar fashion. Nodal proteins are a subfamily of the TGF-β family of signalling proteins that are critical in embryonic development (Schier 2003). Observing the nodal homologs squint (Sqt) and cyclops (Cyc) with FCS demonstrated a similar diffusion coefficient but at a velocity inconsistent to FRAP measurements (Wang et al. 2016). Taken together, these results suggest a higher order of regulation such as degradation and/or sequestering of the ligands that are not uniform across the morphogen gradient but are crucial to its generation, consistent with the proposed ‘source–sink mechanism’ for Fgf8.

The Wnt family of proteins are a further class of morphogen that play an essential role during embryonic development. Wnt3 is often described as a β-catenin-dependent Wnt ligand, which promotes cell survival, proliferation and differentiation phenotypes (Niehrs 2012). Wnt3 expression can be detected in the developing vertebrate brain anlage (Roelink and Nusse 1991; Bulfone et al. 1993; Garriock et al. 2007; Clements et al. 2009; Anne et al. 2013). Mutations in the Wnt3 gene lead to patterning defects of the forebrain, midbrain and cerebellum primordia (Wilson and Houart 2004). The function of Wnt3 has been characterised during brain development in zebrafish (Clements et al. 2009; Mattes et al. 2012). However, like Fgf8, it was previously unknown whether the dynamics of Wnt3 are responsible for the formation of its signalling gradient. Wnt3-GFP dynamics have been measured in the dorsal zebrafish brain anlage in zebrafish in vivo by using line-scanning FCS (lsFCS) (Teh et al. 2015; Ng et al. 2016). The behaviour of Wnt3-GFP molecules can be grouped in different fractions depending on their mobility. These different populations of Wnt3-GFP molecules suggest a number of different transport mechanisms occurring simultaneously through multiple transport mechanisms. While the origins of the different fractions are not immediately clear, the fast intracellular fraction most likely represented recent cytosolic Wnt3-GFP transport to or from the membrane. The slow intracellular fraction might reflect Wnt3-GFP trafficking at the plasma membrane. Conversely, the extracellular fraction highlighted either an aggregation of lipid-modified Wnt3-GFP (Vyas et al. 2008) or formation of complexes between Wnt3-GFP and some extracellular matrix components, such as heparin sulphate proteoglycans (HSPG) (Kleinschmit et al. 2010) or secreted frizzled-related proteins (Mii and Taira 2009). In addition, one cannot rule out that Wnt3-GFP is loaded on larger cargo transporters such as lipoproteins (Neumann et al. 2009), exosomes (Gross et al. 2012) or cytonemes (Stanganello et al. 2015; Mattes et al. 2018). This evidence was further supported by a recent study that compared the diffusion velocity of Wnt3-GFP within the brain anlage of developing zebrafish embryos to secreted GFP and membrane tethered GFP (Veerapathiran et al. 2020). When observed with FCS with or without the presence of HSPGs, it was noted that the diffusion speed of the fast extracellular fraction of Wnt3-GFP increased almost twofold, whereas secreted and membrane tethered GFP controls were unchanged. These findings suggested that the diffusion of Wnt3-GFP was regulated extracellularly by proteins such as HSPGs and that their interactions are crucial to determining their morphogen gradients.

The potential for post-secreted Wnt regulation also plays a significant role in morphogen gradient formation. Wnt ligands obtain hydrophobic characteristic as a result of a post-translational modification of a palmitoyl group from an O-acetyltransferase called Porcupine (Porcn) and two glycosylation groups (Torres et al. 2019). These modified Wnts then associate with the lipophilic subcellular compartments such as the plasma membrane (Yu et al. 2014). Interestingly, as a limitation of in vitro cell-based assays, transiently transfected Wnts remain largely within the endoplasmic reticulum with only a small fraction locating to the cell membrane (Coudreuse and Korswagen 2007; Burrus and McMahon 1995). Therefore, investigations to the membrane localisation and distribution of Wnt3 would benefit greatly with an in vivo system. To observe clustering of Wnt over a large surface area, single-plane illumination microscopy FCS (SPIM-FCS) was devised (Wohland et al. 2010). SPIM-FCS is a multiplexed camera-based imaging FCS-based modality that combines SPIM with fast array detectors to allow simultaneous FCS studies on thousands of adjacent observation volumes (Wohland et al. 2010). This technique generates a spatial map of FCS measurements allowing pinpointing unique interactions within specific locations that may be missed using point-based FCS or lsFCS. The SPIM-FCS analysis in zebrafish showed that Wnt3-GFP can associate with cholesterol-dependant domains on the apical membrane of cerebellar cells. These ordered membrane domains, often generalised as membrane (lipid) rafts, are specialised membrane microdomains that are characterised by dynamic assemblies of saturated lipids, sterols, and lipid-anchored proteins and regarded as highly ordered (Simons and Ikonen 1997; Sezgin et al. 2017a, b, c).

As a result of the success of SPIM-FCS, only a year later it was used to determine the localisation of Wnt receptors on cell membranes (Sezgin et al. 2017a, b, c). Various studies linked also the localisation of the receptors to specific clusters of ‘ordered’ or ‘disordered’ bulk compositions on cell surface membranes, playing specific roles in the interactions between Wnt ligands and their cognate receptors. For example, various (co-)receptors are localised differentially at the plasma membrane depending on the membrane order and so it was theorised that microdomains act as a signalling platform (Özhan et al. 2013; Yamamoto et al. 2008; Sezgin et al. 2017a, b, c). How these domains could direct Wnt binding was previously unresolved; however, FCS measurements provided the means to observe these regions and quantify the binding of Wnt ligands to them. Wnt co-receptors LRP5/6 can be found evenly distributed along the membrane; however, phosphorylation of these receptors—as a result of binding to Wnt—is preferentially found within ordered regions (Yamamoto et al. 2008; Sezgin et al. 2017a, b, c). These results have prompted interest into the targeting of proteins that have preferential localisation to these ordered regions as a therapeutic target for pathologies that depend on aberrant Wnt signalling (Sezgin et al. 2017a, b, c).

As explained above, the implementation of FCS has been crucial in advancing our knowledge for a wide range of morphogens to date. This is, however, by no means the only available optics-based technique that explores molecular behaviour. Techniques ranging from confocal microscopy to single or multiple particle tracking techniques such as FRET (Piston and Kremers 2007), FRAP (Lippincott-Schwartz et al. 2003) or three-dimensional single particle orbital tracking (3D SPT) (Wehnekamp et al. 2019) have also contributed to our understanding of morphogen trafficking. As expected, these alternative techniques explore different aspects of molecular behaviour to draw their conclusions. This inevitably leads to discrepancies of results between the various techniques such as the aforementioned difference in observed morphogen diffusion velocity of nodal and Fgf8 between FRAP and FCS measurements (Wang et al. 2016; Müller et al. 2013). FRAP observes the behaviour of entire populations of fluorescent proteins instead of that of a single or small number of proteins. This technique measures the diffusion velocity of these fluorophores by observing the time taken for surrounding fluorophores to re-populate an area in which most fluorophores have been irreversibly photobleached. In contrast to FCS, which observes single molecules passing through a probe volume of a few femtolitres, FRAP combines the velocity of all fluorophores passing through a multitude of differing environments (e.g., through intra- or extracellular spaces). Therefore, the global effective diffusion coefficient of a population of molecules moving through a tissue measured by FRAP should be expected to be lower than the local diffusivity within a small extracellular volume measured by FCS. Indeed, FCS experiments for secreted GFP in zebrafish embryos yielded a local extracellular diffusion coefficient of ∼ 90 μm^2^/s, which is about double the effective diffusion coefficient of ∼ 40 μm^2^/s measured by FRAP (Yu et al. 2009; Müller et al. 2012). Therefore, both techniques observe and explain the diffusion of the fluorophore but excel in specific niches.

### Fluorescence cross-correlation in zebrafish

In addition to the motion of biomolecules in zebrafish, FCS can also be used to determine interactions between two molecules, for example, to determine binding affinities of ligands and receptors. As described in the previous section, FCCS is the study of correlating the activity of two spectroscopically distinct fluorophores. This process determines the characteristics of the fluorophores, essentially identical to FCS, for each of the two molecular species, but can also determine the ratio of bound to unbound fluorophores (Mütze et al. 2011). This allows us to quantitatively characterise their affinity, quantified by the apparent binding constant *K*_D_, and infer their biological relationship.

One of the first measurements of *K*_D_ within zebrafish using FCCS were used to measure the interaction between the small Rho-GTPase Cdc42 and the IQGAP1, an actin-binding scaffolding protein (Shi et al. 2009a, b). However, due to alternating optical densities within in vivo specimens, different excitation spectra used to excite the POIs would travel through the sample at non-equal beam paths, resulting in reduced signal-to-noise ratio. Instead, single-wave FCCS (SW-FCCS) was developed which uses a single excitation wavelength to excite both fluorophores (Hwang and Wohland 2004, 2005). Using SW-FCCS, a *K*_D_ of 105 ± 11 nM was measured, along with the protein complex percentage being 41.6 ± 9.2% that of a positive control indicates that Cdc42 binds strongly to IQGAP1. When compared to the Cdc42^T17N^ mutant form, this K_D_ increases massively to above 1500 nM showing a weak affinity and therefore loss of binding. Interestingly, while the mutant Cdc42^T17N^ was smaller than IQGAP1 (~ 55 kDa vs 120 kDa, respectively) Cdc^T17N^ diffused more slowly, a phenomenon argued to be as a result of the dominant negative Cdc42^T17N^ forming complexes with other proteins. These results were comparable to that of FCCS experiments executed in CHO cells. However, the constitutively active Cdc42^G12V^ was found to have higher K_D_ to IQGAP1 than that in zebrafish embryos that was suggested to be a Ca^2+^-dependency interaction of IQGAP1 to other effectors such as F-actin that would bind in competition to Cdc42^G12V^ (Shi et al. 2009a, b). Several reports have shown that binding of Ca^2+^/Calmodulin to IQGAP1 reduces affinity to Cdc42 and F-actin, a phenomenon that is not replicated in vitro and, therefore, improperly reflects physiological activity (Ho et al. 1999; Mateer et al. 2002). An additional explanation of the discrepancy between the FCS studies was explained by the penetration depth of FCS in zebrafish embryos resulting in a widening of the focal volume, which increases the *K*_D_ recorded. This, along with a distortion in long wavelength beams in comparison with the shorter wavelength beams, results in an overall over-estimation of the dissociation and *K*_D_ measured.

Similar to Cdc42 and IQGAP1, many proteins studied under FCCS in vivo found discrepancies in their binding affinities when compared to in vitro. From this, FCCS has been implemented for a wide range of protein candidates from morphogens such as FGF and Wnt to structural extracellular proteins such as cadherins. In vitro analysis on FGF receptor–ligand interaction identified multiple FGF ligand binding partners to multiple FGF receptors (FGFR) at variable degrees of activity. FGF8 was observed an estimated 20-fold increase in activity with FGFR4 than it does with FGFR1 (Ornitz et al. 1996). Dual-colour scanning FCCS across the membrane of gastrulating zebrafish embryos determined that while the affinity for FGF8 remained significantly higher for FGFR4 over FGFR1 in vitro, this effect was only twofold in vivo (Ries et al. 2009). The differences between in vitro and in vivo data was postulated to be interactions of extracellular matrix molecules—such as HSPGs—that directly or indirectly modulated receptor–ligand interactions that are lacking from the cell-based in vitro assays (Hou et al. 2007). Furthermore, Cadherin 2, a cell surface adhesion protein, demonstrated differing results between in vitro and in vivo studies. Studies with FCCS in vitro found that the binding of Cadherin 2 to soluble Cadherin2 ectodomains was in the range of 80 ± 20 μM and 720 μM (Häussinger et al. 2004). This experiment was repeated in vivo in the mesenchymal cells of the zebrafish presomitic mesoderm which found intercellular homotypic binding of Cadherin 2 on adjacent cells was 200 ± 100 nM, a far tighter *K*_D_ than that found in vitro (Jülich et al. 2015). Furthermore, cross-correlation between Integrin α5β1 heterodimers expressed on the surface of adjacent cells revealed an apparent *K*_D_ of 750 ± 100 nM. It was argued that the discrepancies between the findings resulted from constrained anti-parallel arrangement of cadherins in the membranes of adjacent cells, a parameter that could not be recapitulated in solution-based studies.

As the primary objective for morphogen ligands is to bind to their cognate receptor to elicit their respective signal pathways, FCCS studies have been implemented to observe these binding affinities. As explained previously, the family of nodal ligands is comprised of several homologs such as Sqt and Cyc (Schier 2003). Sqt and Cyc are known to operate at different distances from the source cell; however, it was unclear whether factors such as affinity to receptors or inhibitors among others were crucial to their morphogen gradient (Chen and Schier 2001; Jing et al. 2006; Müller et al. 2012; Tian et al. 2008). Sqt in general has peak activity further from the source cell than Cyc and so was hypothesised to be in part due to a reduced affinity for the same receptors such as Acvr2b. Simultaneously, soluble inhibitors such as Lefty prevent nodal binding to Acvr2b and were suspected to also play a role. Surprisingly, it was determined that Sqt bound with a higher affinity to both Lefty and Acvr2b than Cyc, by almost a factor of two. These results, taken together with the diffusion coefficient and stability of the ligands, provided conclusive evidence in support for the RCM and ‘source–sink mechanism’ model postulated for Fgf8 (Scholpp and Brand 2004; Yu et al. 2009). The parameters gained from these results were used to generate an in silico model for nodal diffusion which accurately recapitulated the morphogen gradient observed in vivo.

In certain exceptions, protein–protein investigations are limited or fail due to unsuitable conditions in solution or cell-based in vitro assays, for example, cell protrusions through the ECM. Utilising in vivo samples would therefore be necessary in such situations (Fig. 2). Recent evidence suggests that Wnt/PCP signalling influences the formation of filopodia. During zebrafish gastrulation, Wnt11 activates the β-catenin independent Wnt/PCP receptor Ror2 to regulate complex cell migratory processes known as convergence and extension including filopodia generation (Bai et al. 2014). FCCS studies were used to analyse the interaction between Ror2 and another Wnt ligand, here Wnt8a. Wnt8a is considered as β-catenin-dependent Wnt ligand. However, a high cross-correlation amplitude indicates co-diffusion of bound Ror2-mCherry and Wnt8a-GFP and further experiments demonstrated that Wnt8a/Ror2 can activate the β-catenin-independent Wnt/PCP signalling pathway and thus promote filopodia formation (Mattes et al. 2018). Based on the FCCS data, a further analysis showed that these filopodia are Wnt8a/Ror2-bearing protrusions—known as cytonemes—regulating extracellular Wnt dispersal (Zhang and Scholpp 2019; Mattes et al. 2018).

Overall, these examples highlight the differences between in vitro and in vivo FCS analysis through fundamental differences in their biological environment. Whether investigating intracellular, extracellular or transport mediated protein dynamics, we observe different readouts between the two models that support a greater need for in vivo analysis.

### Considerations to zebrafish sample preparation for FCS

Sample preparation of the zebrafish embryo is paramount to successful data acquisition in FCS studies and therefore requires thorough planning and execution. Multiple factors from imaging time post-fertilisation, generation of fluorophores and choice of FCS strategy must be chosen in advance of the study. The majority of these decisions are determined by the hypothesis that is to be tested. These technical decisions will be broadly assessed below with respect to their advantages and disadvantages and their limitations. Further in-depth technical preparation and protocols can be found in a recent review (Ng et al. 2018).

### Generation and application of fluorescently tagged proteins

**Fig. 3:**
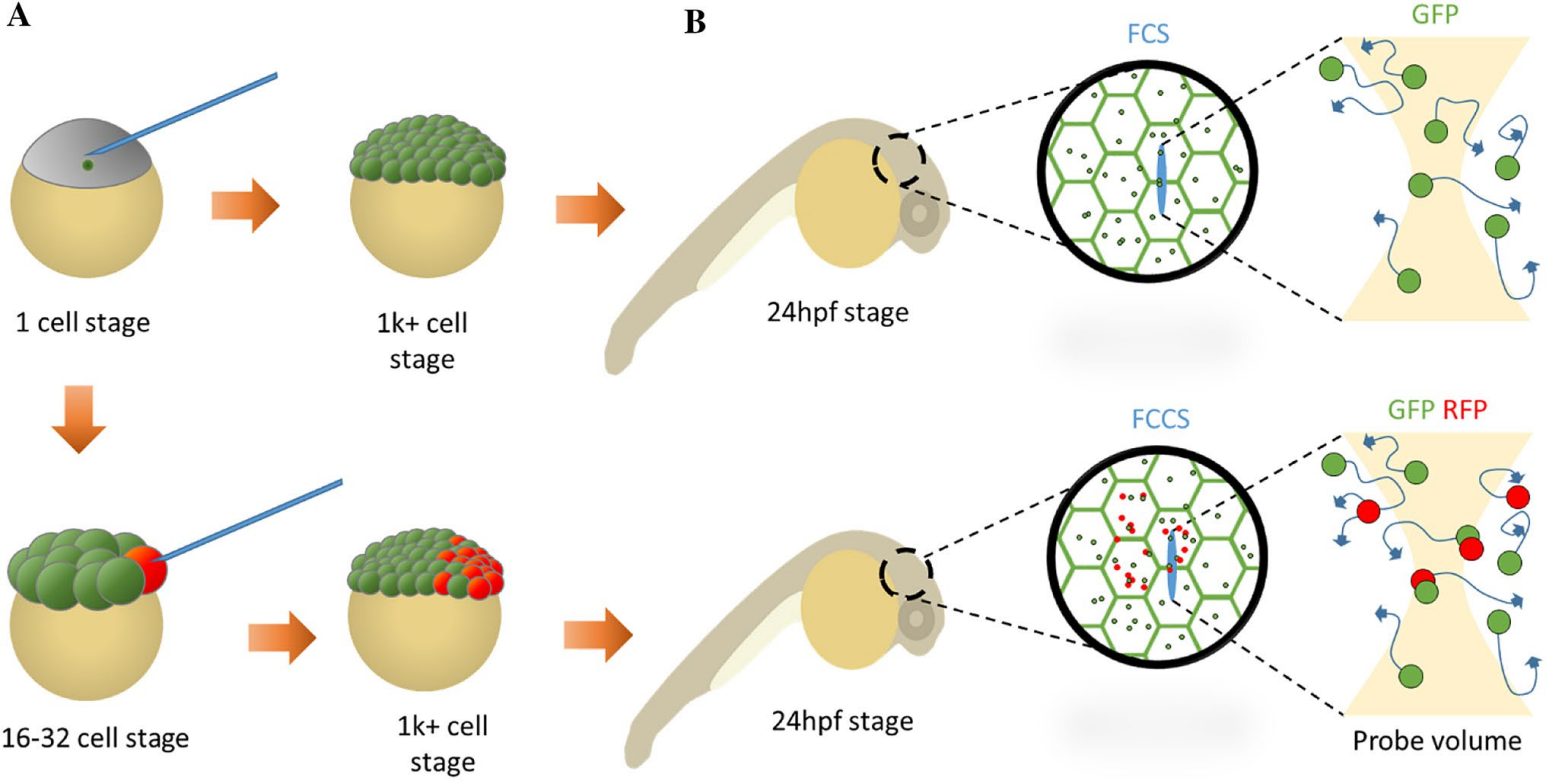
mRNA injection time determines distribution of fluorophore. **a** Microinjection at very early embryo stage (1–4 cell) generates homogenous expression of fluorophore across entire embryo, while later stage injections (16–32 cell) generates confined/mosaic patterning of fluorophore. **b** Depending on time of injection, patterning on embryo can be imaged with FCS or with FCCS using two or more fluorophores

The fluorophores are essential markers of the POI under study. There are several methods used for generating these fluorophores with respective advantages and disadvantages. In zebrafish, the most common method is the generation of fluorescent protein fusion recombinant plasmid DNA constructs and the generation of capped mRNA from these plasmids (Peterson and Freeman 2009; Linney et al. 2004). mRNA microinjection has the benefit of delivering the construct at any stage of the developing embryo as portrayed in Fig. 3. This can be used to track the POI through FCS and/or to observe a phenotypic change using mutant constructs. Expression of the construct is uniform across the population of cells that receive the mRNA and is generally over-expressed, causing saturation of the construct. Over-expression can cause a problem for FCS as it over-saturates the probe volume and is therefore tightly controlled using consistent amount of mRNA for each injection.

An alternative method is the direct modification of the zebrafish genome to allow expression of the gene of interest at the endogenous expression sites at physiological levels. A specific GFP integration (knock-in) can be achieved with a variety of methods from zinc-finger nucleases (ZNFs) (Durai et al. 2005), transcription activator-like effector nucleases (TALENs) (Cermak et al. 2011) and a system based on the prokaryotic clustered, regularly interspaced short palindromic repeats (CRISPR) and the CRISPR associated proteins (Cas) (Jinek et al. 2012). While knock-in technology in fish stock is an attractive option, the process is lengthy and technically intense. The F0 generation must develop into full adults which takes a minimal of 3 months to achieve. After two rounds of crosses including identification of the carriers, the F3 generation can then be analysed (Kimmel et al. 1995; Parichy 2008).

Further considerations must be approached in regard to the fluorophore that is required and the effect this has on the POI. There is a wide selection of fluorophores available, but specific fluorophores are better fit than others. For example, when deciding on fluorophores used in an FCCS study, the two fluorophores must be spectrally distinct to the point of minimal crosstalk (Mütze et al. 2011). Fluorophores emit a range of emission wavelength with the intended emission band ideally being the most intense. This is especially significant for FCCS studies as bleed through of signal from one fluorophore into the other detection channel can be miss-interpreted as co-localisation of the two fluorophores where there is none. It would appear that using fluorophores with the largest difference in peak emission wavelengths would be most favourable, for example, a mCherry tag (610 nm λ_em_) (Merzlyak et al. 2007) with an EBFP tag (448 nm λ_em_) (Subach et al. 2008). However, further problems persist such as poor photostability of certain fluorophores. More commonly, a compromise between emission spectrum overlap and photostability is employed alongside adaptations of FCS modalities that help reduce these limitations such as SW-FCCS or two-photon FCS (TP-FCS).

Fluorescent proteins are themselves large constructs with GFP being 27 kDa that consists of a beta-sheet barrel structure of 4.2 nm (40 Å) in length and 2.4 nm (25 Å) in diameter (Remington 2011). For many large proteins, the size of the fluorophore may pose little to no effect on protein dynamics. However, there remains the possibility of interference from steric hindrance posed by the fluorophore or the unintended cleavage of the fluorophore from the fusion protein if placed before a signalling peptide sequence for example. In the study of ligands, the bulk of the ligated fluorophore can be a serious limitation which risks the improper function or translocation of the ligand.

Lastly, timing of the microinjection of mRNA determines the distribution of the fluorophore which can be used to experimental advantage. Injection at the one cell stage ensures even distribution and expression of the fluorophore, whereas injection later in development will restrict its distribution. This can be useful when local phenotypic changes are observed to be compared to regions of the blastula that are not exposed to the expressed construct. This can be visualised when combined with a dye to highlight the location of the injection later in development and the exact distribution of the construct (Ng et al. 2018).

### Mounting of embryo

Proper mounting is key to ensuring correct alignment of the FCS beam path. Depending on the timing of the experiment, sample mounting can be performed in different ways. For any stage of embryonic development, the zebrafish egg is dechlorinated to remove the chorion—an acellular envelope that surrounds the embryo to protect it from the environment during development (Westerfield 2000). The chorion itself is of no specific use in FCS research and is instead further tissue that obstructs the beam path and owes to further light scatter (Thisse and Thisse 2008). For much later stages (> 3 dpf), this step is unnecessary as the larvae hatch from the chorion themselves, but it can still constitute an unwelcome obstacle for microinjection of mRNA early in the embryo’s development. For very early stage embryos of up to 20 hpf, the embryos are typically mounted using low-melting point agarose immediately after injection without further steps. Beyond this stage, the zebrafish may move with involuntary muscle contraction which must be subdued with the use of anaesthetics such as 0.05% (w/v) tricaine to avoid motion artefacts in FCS experiments. Embryos that are examined at much later stages must be incubated with 0.003% (w/v) PTU at 20–30 hpf to inhibit melanin formation and therefore pigmentation (Ng et al. 2018). The position of the embryo within the agar is crucial to the location the excitation spectra crosses. For example, studying the brain ventricle of the embryo requires the dorsal side of the embryo to be in contact with the cover slip to ensure that it is as close to the objective as possible.

### Concluding remarks

FCS and FCCS have seen a greater rise within the previous decade revealing aspects of flow velocities, protein–protein binding, ligand–receptor affinity and transport and the composition of ECM in the zebrafish. These studies have highlighted crucial differences in results when experiments are repeated in vivo rather than in vitro and demonstrated a clear need for further transition into the in vivo system for future investigations. While many in vivo model organisms exist, this review highlights the advances specifically within the zebrafish model to critically analyse the advancements thus far.

Indeed, it would be unfair to simply view FCS and FCCS as a means to observing protein dynamics alone. As observed with measuring flow velocities, FCS can be adapted and applied to measure a larger range of variables including membrane dynamics, signal mediated clustering and differential protein concentrations across a distance. Interestingly, in the field of Wnt morphogens gradients, we have yet to visualise the changes in morphogen gradient across several cell diameters despite the overwhelming evidence supporting its existence. As SPIM-FCS can resolve the concentration of fluorophores at different locations simultaneously, it could serve as the perfect tool to highlight this phenotype in vivo and potentially reveal further insight into morphogen gradient formation and maintenances. A similar non-standard usage of FCS could be investigated in the assembly and disassembly of protein complexes in vivo. It has been demonstrated that several fractions of diffusing fluorophores are present which are hypothesised to result from interactions with factors such as bulky protein complexes. Protein complexes could be observed in this way by identifying slowly diffusing fractions and determining their magnitude and concentration. In the field of signalling, there is significant evidence of the formation of a receptor–ligand complexes, necessary for transducing the ligands signal intracellularly. FCS and or FCCS experiments could be applied to resolve these complexes and observe their clustering in vivo at the moment of ligand binding.

In conclusion, the observed protein dynamics between in vitro and in vivo assays appear to arise due to fundamental differences between the two models. As it stands at present, although in vitro assays are critical for early protein investigation, the in vivo models are the most physiologically relevant platform for any biochemical analysis. The different results obtained between the two models suggest that a greater use of in vivo analysis must be prioritised. The high-demand for in vivo studies by FCS requires the development of more model organisms primed for these measurements like the zebrafish embryo. Considering the multi-functional uses of FCS and the possibilities to measure interactions of biological macromolecules in a multitude of subcellular environments in a living animal, we are now in a position to describe the dynamic processes operating in a living cell with a high accuracy.
